# Retraction Note: Annonacin promotes selective cancer cell death via NKA-dependent and SERCA-dependent pathways

**DOI:** 10.1038/s41419-022-05218-5

**Published:** 2022-09-20

**Authors:** Andreas Yiallouris, Ioannis Patrikios, Elizabeth O. Johnson, Evangelia Sereti, Konstantinos Dimas, Cristian De Ford, Natalia U. Fedosova, Wolfgang F. Graier, Kleitos Sokratous, Kyriakos Kyriakou, Anastasis Stephanou

**Affiliations:** 1 School of Medicine, European University Cyprus, Nicosia, Cyprus; 2 School of Medicine, National and Kapodestrian University of Athens, Athens, Greece; 3 Department of Pharmacology, School of Health Sciences, University of Thessaly, Volos, Greece; 4 Department of Pharmaceutical Biology and Biotechnology, Spemann Graduate School of Biology and Medicine (SGBM), Albert Ludwigs University Freiburg, Freiburg, Germany; 5 Department of Biomedicine-Physiology and Biophysics, Aarhus University, Aarhus, Denmark; 6 Gottfried Schatz Research Center, Molecular Biology & Biochemistry, Medical University of Graz, Graz, Austria; 7 Department of EM/Molecular Pathology, Institute of Neurology and Genetics, Nicosia, Cyprus

Retraction to: *Cell Death and Disease* 10.1038/s41419-018-0772-x, published online 9 July 2018

The authors have retracted this article because of concerns about the reliability of the data presented in Figure 4A and Figure 4B.

Anastasis Stephanou, Andreas Yiallouris, Ioannis Patrikios, Elizabeth O. Johnson, Konstantinos Dimas, Cristian De Ford' Natalia U. Fedosova, Wolfgang F. Graier, Kleitos Sokratous and Kyriakos Kyriakou agree with this retraction. Evangelia Sereti did not respond to correspondence from the Publisher about this retraction.

